# Evaluation of Collagenic Porcine Bone Blended with a Collagen Gel for Bone Regeneration: An In Vitro Study

**DOI:** 10.3390/ijms26157621

**Published:** 2025-08-06

**Authors:** Tania Vanessa Pierfelice, Chiara Cinquini, Morena Petrini, Emira D’Amico, Camillo D’Arcangelo, Antonio Barone, Giovanna Iezzi

**Affiliations:** 1 Department of Medical, Oral and Biotechnological Sciences, University “G. d’Annunzio”, Via dei Vestini 31, 66013 Chieti, Italy; tania.pierfelice@unich.it (T.V.P.); morena.petrini@unich.it (M.P.); camillo.darcangelo@unich.it (C.D.); gio.iezzi@unich.it (G.I.); 2 Department of Surgical, Medical, Molecular Pathologies and of the Critical Area, University of Pisa, Lungarno Antonio Pacinotti, 43, 56126 Pisa, Italy; chiara.cinquini@gmail.com (C.C.); barosurg@gmail.com (A.B.); 3 Dental Biomaterials Research Unit, University of Liege, 62063 Liege, Belgium

**Keywords:** collagenic biomaterial, bone graft, collagen gel, bone regeneration, alveolar bone augmentation

## Abstract

A thermosensitive collagen-based gel (TSV gel), containing type I and III collagen, has been developed to improve the handling and stability of bone graft materials. However, its direct effect on osteoblasts is not well understood. This in vitro study evaluated the biological response of human oral osteoblasts to four bone substitutes: OsteoBiol^®^ GTO^®^ (larger granules with 20% TSV gel), Gen-OS^®^ (smaller granules), Gen-OS^®^ combined with 50% TSV gel (Gen-OS^®^+TSV), and TSV gel alone. Cell proliferation, adhesion, morphology, collagen and calcium deposition, alkaline phosphatase (ALP) activity, gene expression of osteogenic markers and integrins, and changes in pH and extracellular calcium and phosphate levels were investigated. All materials supported osteoblast activity, but Gen-OS+TSV and GTO showed the most pronounced effects. These two groups promoted better cell adhesion and proliferation, higher ALP activity, and greater matrix mineralization. GTO improved cell adhesion, while the addition of TSV gel to Gen-OS enhanced biological responses compared with Gen-OS alone. Integrins α2, α5, β1, and β3, important for cell attachment to collagen, were notably upregulated in Gen-OS+TSV and GTO. Both groups also showed increased expression of osteogenic markers such as BMP-2, ALP, and osteocalcin (OCN). Higher extracellular ion concentrations and a more alkaline pH were observed, particularly in conditions without cells, suggesting active ion uptake by osteoblasts. In conclusion, combining TSV gel with collagen-based granules improves the cellular environment for osteoblast activity and may support bone regeneration more effectively than using either component alone.

## 1. Introduction

The management of alveolar bone atrophy represents a significant challenge in implant dentistry. Clinical limitations include insufficient bone volume and quality, prolonged treatment times, risk of graft failure, complex surgical procedures, and the high cost of regenerative techniques. Bone graft materials are employed to promote bone regeneration and repair. The use of biomaterials in techniques such as guided bone regeneration (GBR) aims to restore bone volume and quality, thereby supporting implant rehabilitation. Bone grafts offer structural support and act as a framework for cell growing to form bone [1]. They can be classified as autografts (from the patient), allografts (from a human donor), xenografts (from animals), or alloplasts (synthetic materials) [2]. Among xenografts, porcine-derived scaffolds have shown promising results in several clinical trials. These materials demonstrate good osteoconductive properties, minimal inflammatory response, and effective bone regeneration with limited residual graft material [3,4,5]. While numerous graft materials are commercially available, they differ in biological behavior and clinical outcomes. Collagenic bone substitutes seem to have more advantages than non-collagenic ones [6,7]. In a recent study, Miyauchi et al. demonstrated that both non-collagenic and collagenic xenografts promoted new bone formation in sinus lift procedures. However, the collagenic xenografts exhibited greater biomaterial resorption, which led to more pronounced volumetric shrinkage of the augmented area and a higher content of newly formed bone in the regions close to the bone walls [7]. Another study has confirmed the effectiveness of porcine-derived cortico-cancellous collagenic grafts in preserving alveolar ridge dimensions following tooth extraction [8]. Despite their benefits, bone substitutes are most commonly used in granule form, which can lead to collapse or compression within the defect site, potentially compromising bone regeneration [9]. To overcome this limit, several stabilizing agents have been produced. Recently, a thermosensitive gel (TSV gel) containing type I and III collagen was developed with the purpose of providing mechanical stability to bone substitutes and barrier membranes. Collagen plays a crucial role in wound healing, serving as a scaffold for cell adhesion, migration, and proliferation, while also supporting angiogenesis and tissue repair [10,11]. Furthermore, a study reported that the presence of collagen could play a role in the mechanism of reabsorption of particles [12]. In several studies, collagenic bone substitutes have different compositions and granulometry; however, it is not clear whether these factors and the use of a thermogel can affect the response of osteoblasts. Therefore, in this study, human oral osteoblasts (hOBs) were cultured with differently sized collagenic bone substitutes (OsteoBiol^®^ GTO^®^ and OsteoBiol^®^ Gen-OS^®^) and with OsteoBiol^®^ TSV gel.

In this study, an in vitro model of primary oral osteoblasts (hOBs) was used to compare the effects on the osteoblastic activities of four TEST groups (GTO, Gen-OS, TSV gel, Gen-OS+TSV) and untreated cells as a control group (CTRL). The evaluated osteoblastic activities were cell proliferation, morphology, adhesion, collagen and calcium deposition, alkaline phosphatase (ALP) activity, and gene expression of osteogenic markers and integrins. Furthermore, given the high sensitivity of osteoblasts to their biochemical microenvironment, and considering that the bone mineralization occurs within the collagen matrix and is facilitated by extracellular ions such as calcium and phosphate [13,14], in this study, the impact of these biomaterials on pH levels and calcium and phosphate ion concentrations in the extracellular environment was evaluated. The hypothesis of this study is that different biomaterials modulate the osteogenic behavior of oral osteoblasts to varying degrees, potentially by altering both the local biochemical environment and cellular function. Therefore, the objectives of this study were (i) to compare the osteoblastic responses across different biomaterial groups; (ii) to assess how each material affects key osteogenic markers and extracellular matrix formation; and (iii) to evaluate the influence of biomaterials on extracellular pH and ionic composition relevant for mineralization.

## 2. Results

### 2.1. Viability

**Figure 1:**
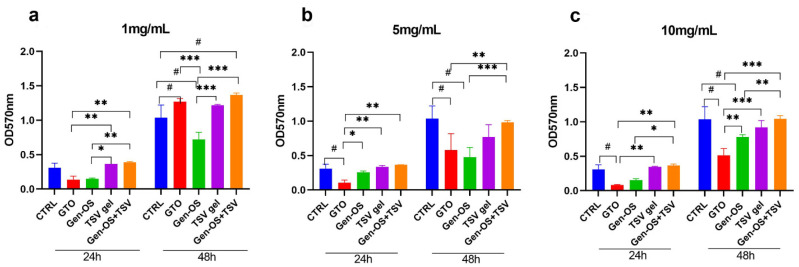
Cell viability assay after 24 h and 48 h of incubation with various biomaterials at different concentrations: (**a**) 1 mg/mL, (**b**) 5 mg/mL, (**c**) 10 mg/mL. The values on the histograms are expressed in terms of optical density (OD) as the mean ± SD. Statistical significant differences are reported in terms of the cell growth of treated groups compared with the untreated group (^#^ *p* < 0.05) or each other (* *p* < 0.05; ** *p* < 0.01; *** *p* < 0.001). All data were obtained from three independent experiments, each performed in triplicate.

At 1 mg/mL, TSV gel and Gen-OS+TSV groups showed a significant difference (*p* < 0.05) with respect to Gen-OS and GTO after 24 h. Cell viability in Gen-OS+TSV, GTO, and TSV gel were higher than the control after 48 h. Gen-OS+TSV and GTO showed comparable values (Figure 1a). At 5 mg/mL, the histogram showed a similar trend at 24 and 48 h. Gen-OS+TSV showed comparable proliferation levels with CTRL at both times (Figure 1b). A significant decrease was observed for GTO and Gen-OS compared with CTRL (*p* < 0.05) and compared with Gen-OS+TSV (*p* < 0.001). At 10 mg/mL, Gen-OS+TSV displayed viability levels similar to CTRL at both experimental times (Figure 1c). TSV gel showed a slight reduction in cell growth after 48 h. GTO and Gen-OS groups exhibited a strong decrease compared with CTRL (*p* < 0.05) and to Gen-OS+TSV (*p* < 0.001) at both experimental times. Based on these results, 1 mg/mL was chosen for the next experiments.

### 2.2. Morphology and Adhesion

**Figure 2:**
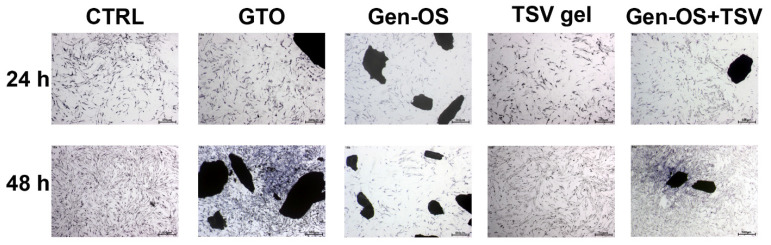
Microscopic images with Toluidine Blue staining depicting the morphological changes of osteoblasts treated with the bone substitutes. The images were taken at 24 and 48 h post-treatment. (Magnification: 18×—scale bar: 500 µm). All data were obtained from three independent experiments, each performed in triplicate.

The overall morphology of the cells, characterized by an elongated, spindle-like shape, was similar across all groups; however, differences were observed in adhesion and spread (Figure 2). After 48 h, in the Gen-OS group, cells did not form a homogeneous layer, while in the TSV gel group, hOBs were well spread. The Gen-OS+TSV and GTO groups demonstrated the most favorable adhesion among all experimental groups. Notably, cells treated with Gen-OS+TSV and GTO showed strong attachment to granules, indicating enhanced integration with the bone grafting material.

### 2.3. Collagen I Deposition

**Figure 3:**
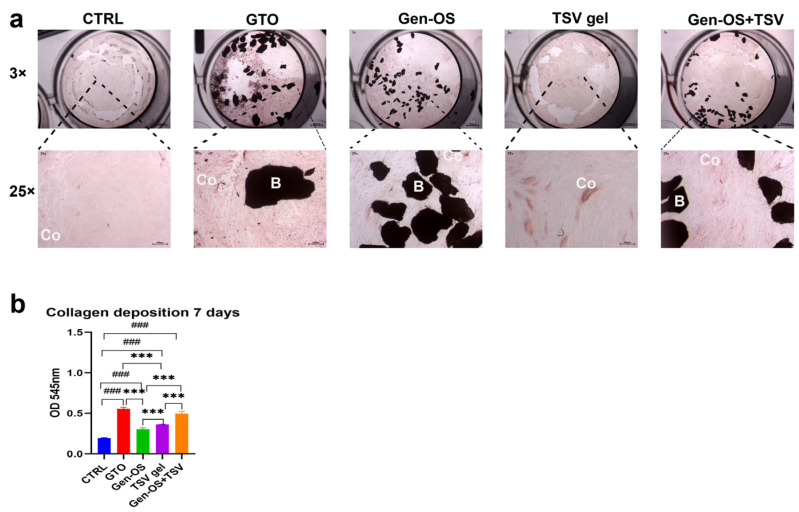
(**a**) Representative images of Picro-Sirius Red staining demonstrating collagen deposition (red) in different treatment groups. (**b**) Collagen deposition was measured at 7 days post-treatment. Data are reported as the mean ± SD. Statistical significance was determined using an ANOVA test followed by Tukey’s post hoc test. *** *p* < 0.001among different groups. ^###^ *p* < 0.001 compared with CTRL (B: biomaterial; Co: collagen deposition) (magnification 3×—scale bar: 500 µm, magnification 25×—scale bar: 300 µm). All data were obtained from three independent experiments, each performed in triplicate.

Microscopic images (Figure 3a) revealed minimal collagen deposition in CTRL, moderate collagen deposition in Gen-OS and TSV gel, and substantial deposition in GTO and Gen-OS+TSV groups after 7 days. In addition, Gen-OS+TSV and GTO groups showed the most organized collagen network. The collagen deposition quantification (Figure 3b) confirmed that all treated groups significantly increased collagen levels compared with CTRL. The GTO group exhibited the highest collagen deposition. Gen-OS and TSV gel had a moderate but still significantly higher effect (*p* < 0.001) with respect to CTRL. Gen-OS+TSV and GTO treatments showed a significant increase in deposition in relation to Gen-OS and TSV gel (*p* < 0.001).

### 2.4. Mineralization

**Figure 4:**
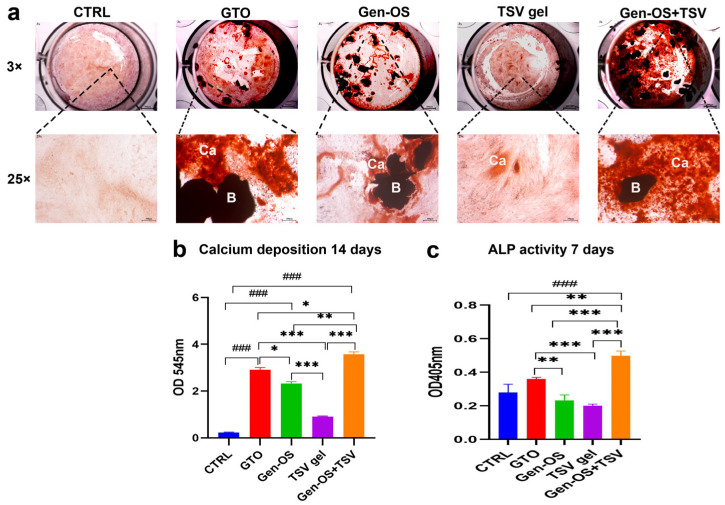
Effect of bone substitutes on ALP activity and calcium deposition in bone tissue. (**a**) Representative images of Alizarin Red staining after 14 days. (**b**) Calcium deposition quantization of 14 days. (**c**) ALP activity was measured after 7 days of treatment. Data are presented as the mean ± SD. Statistical significance was determined by an ANOVA test followed by Tukey’s post hoc test. ^###^ *p* < 0.001 compared with CTRL; * *p* < 0.05, ** *p* < 0.01, *** *p* < 0.001 in relation to the other test groups (B: biomaterial, Ca: calcium nodules) (magnification 3×—scale bar: 500 µm, magnification 25×—scale bar 300 µm). All data were obtained from three independent experiments, each performed in triplicate.

Calcium deposition was evaluated at 14 days using ARS staining and quantified with CPC (Figure 4a,b). Microscopic examination of mineralization via staining revealed differences in calcium deposition among the groups (Figure 4a). The GTO, Gen-OS, and Gen-OS+TSV groups showed an intense staining with the presence of calcium nodules that were more pronounced in Gen-OS+TSV followed by GTO. The quantitative analysis confirmed the microscopic observations (Figure 4b). The Gen-OS+TSV group showed the highest calcium deposition levels. The GTO and Gen-OS groups also exhibited significantly higher calcium deposition than CTRL and TSV gel (*p* < 0.001). In contrast, TSV gel displayed moderate calcium deposition levels, which were significantly lower than other test groups but higher than CTRL.

To assess the early osteoblastic functions, alkaline phosphatase (ALP) enzymatic activity was measured at day 7 (Figure 4c). The Gen-OS+TSV group exhibited the highest ALP value (*p* < 0.001), followed by the GTO group. Both groups showed significantly increased levels with respect to Gen-OS (*p* < 0.01), TSV gel (*p* < 0.001) and CTRL (*p* < 0.001).

### 2.5. Ions in the Extracellular Environment

**Figure 5:**
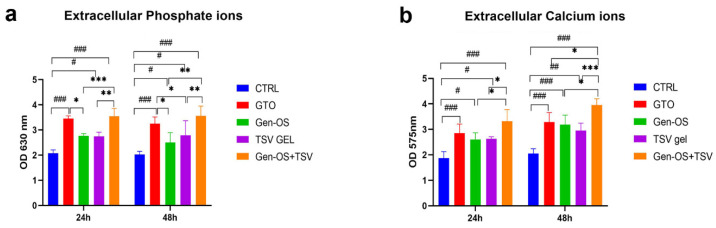
Quantification of extracellular phosphate (**a**) and calcium (**b**) ion levels in the absence of cells. Bone substitutes (GTO, Gen-OS, TSV gel, Gen-OS+TSV) were incubated in the medium for 24 and 48 h. Data are reported as the mean ± SD. *** *p* < 0.001, ** *p* < 0.01, * *p* < 0.05 compared with the other test groups and with the control; ^#^ *p* < 0.05, ^##^ *p* < 0.01, ^###^ *p* < 0.001. All data were obtained from three independent experiments, each performed in triplicate.

**Figure 6:**
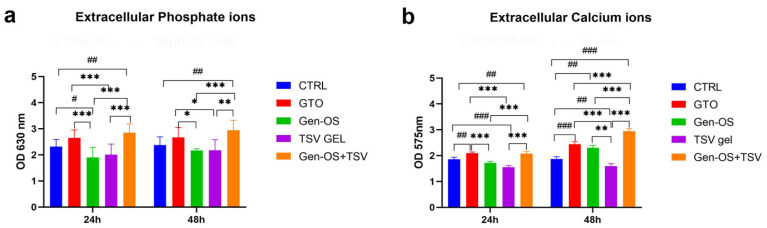
Quantification of extracellular phosphate (**a**) and calcium (**b**) ion levels in the cell culture. hOBs were treated with bone substitutes (GTO, Gen-OS, TSV gel, Gen-OS+TSV) at 24 and 48 h. Data are reported as the mean ± SD. *** *p* < 0.001, ** *p* < 0.01, * *p* < 0.05 compared with the other test groups and with the control ^#^ *p* < 0.05, ^##^ *p* < 0.01, ^###^ *p* < 0.001. All data were obtained from three independent experiments, each performed in triplicate.

Phosphate and calcium ions were quantified in the extracellular environment, both in the absence (Figure 5) and presence of cells (Figure 6), after 24 and 48 h. In the absence of cells, phosphate ions were significantly higher in GTO and Gen-OS+TSV compared with the control and other groups at both time points, with Gen-OS+TSV showing the highest values. All treated groups exhibited increased phosphate release relative to CTRL (Figure 5a). Similarly, the Gen-OS+TSV group demonstrated the highest calcium concentrations at both 24 and 48 h, followed by GTO and Gen-OS, which also showed significantly elevated levels compared with the control (Figure 5b).

In the presence of cells, at 24 h and 48 h, the GTO and Gen-OS+TSV groups showed higher levels of phosphate ions compared with the CTRL, Gen-OS, and TSV gel groups (Figure 6a). GTO and Gen-OS+TSV exhibited comparable levels of phosphate ions release. The extracellular calcium ions showed a similar trend. In detail, the Gen-OS+TSV and GTO groups demonstrated a statistically relevant change in the calcium ions level with respect to CTRL after 24 h (*p* < 0.01) and 48 h (*p* < 0.001). At 24 h, GTO and Gen-OS+TSV exhibited comparable levels of calcium ions release. Moreover, the Gen-OS+TSV group exhibited the highest calcium ion release at 48 h (Figure 6b). Interestingly, the extracellular environment containing only biomaterials exhibited considerably higher concentrations of phosphate and calcium ions than when osteoblasts were also present.

### 2.6. Changes in Medium pH During Cell Culture

Table 1 reports pH measurements of the culture media containing the biomaterials at a concentration of 1 mg/mL, in the absence of cells. These measurements were performed at 24 and 48 h to assess the direct effect of each material on the extracellular environment. At 24 h, pH values ranged from 7.46 to 9.06, while at 48 h, they ranged from 7.46 to 8.62.

Table 2 shows the pH measurements of the culture media collected from osteoblast cultures treated with the same concentration (1 mg/mL) of each biomaterial. These values reflect the combined effect of the biomaterials and cellular activity. At 24 h, pH values ranged from 7.74 to 9.11, while at 48 h, they ranged from 8.01 to 8.62. In both conditions and at both time points, the Gen-OS+TSV group showed the highest alkalinization of the medium.

### 2.7. Gene Expression

**Figure 7:**
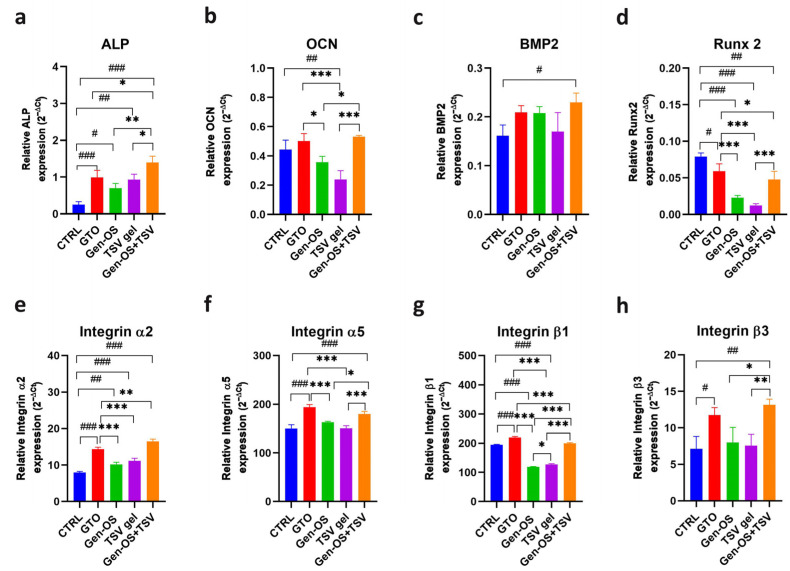
Relative mRNA expression of osteogenic markers (**a**) ALP, (**b**) OCN, (**c**) BMP2, (**d**) Runx2 and integrins subunits (**e**) integrin α2, (**f**) integrin α5, (**g**) integrin β1, (**h**) integrin β3 after the treatment with bone substitutes. Gene expression was normalized to βACT. Data are reported as the mean ± SD. Statistical significance was determined using an ANOVA test followed by Tukey’s HSD test. *** *p* < 0.001, ** *p* < 0.01, * *p* < 0.05 compared with the other test groups; ^#^ *p* < 0.05, ^##^ *p* < 0.01, ^###^ *p* < 0.001. All data were obtained from three independent experiments; each performed in triplicate.

The expression of osteoblastic activity-related genes and integrins is shown in Figure 7. ALP mRNA levels were significantly upregulated in all treated groups relative to the control, with Gen-OS+TSV showing the highest expression followed by GTO, TSV gel, and Gen-OS (Figure 7a). OCN expression was highest in the Gen-OS+TSV group, followed by GTO, with lower levels observed in all other groups.

GTO and Gen-OS+TSV showed comparable levels of OCN expression (Figure 7b). BMP2 expression significantly increased in Gen-OS+TSV compared with CTRL (*p* < 0.05). GTO and Gen-OS appeared upregulated compared with CTRL. TSV gel exhibited comparable expression levels with CTRL. No statistical difference between Gen-OS+TSV and GTO was observed (Figure 7c). Runx2 expression was notably downregulated in all tested groups compared with CTRL, and a heavy downregulation was observed for TSV gel (Figure 7d).

Integrins α2, α5, β1, and β3 (Figure 7e–h) were upregulated in Gen-OS+TSV compared with the control. A significant increase in all the integrins analyzed was also observed in GTO relative to CTRL. Gen-OS+TSV exhibited the most robust effects on integrins α2 and β3 compared with the other groups (Figure 7e,h), while GTO exhibited the most robust effects on integrins α5 and β1 with respect to other groups (Figure 7f,g). Gen-OS and TSV gel showed similar levels to CTRL, except for integrin β1, which was downregulated.

## 3. Discussion

The application of collagen-based biomaterials in alveolar bone augmentation is currently a field of great interest [15]. In this study, an in vitro culture of oral osteoblasts was used to investigate the extracellular environment and the activities of cells seeded with different collagenic bone substitutes (OsteoBiol^®^ GTO^®^, OsteoBiol^®^ Gen-OS^®^, OsteoBiol^®^ TSV gel, OsteoBiol^®^ Gen-OS^®^+TSV). This study showed that all tested collagenic bone substitutes had positive effects on osteoblastic activities, but the combination of TSV gel with biomaterial granules demonstrated the most significant osteogenic potential. Osteoblasts cultivated with Gen-OS+TSV and GTO were elongated and had spindle-like morphologies with good attachment to the particles. It has been shown that the adherence of osteoblasts to bone graft materials is necessary for new bone formation [16]. In this study, cell proliferation and adhesion were significantly higher in Gen-OS+TSV and GTO than in TSV gel and Gen-OS. The particle size could be an influencing factor. An in vitro study on osteoblasts cultured with different bone grafts showed the better adherence of cells for larger particles [17]. In the present study, osteoblasts adhered more to GTO particles that exhibit larger granules than Gen-OS particles. However, when Gen-OS was mixed with TSV gel, the attachment of osteoblasts was enhanced. This suggested that the presence of collagen gel in Gen-OS+TSV and GTO groups provided a more favorable microenvironment for osteoblasts. Collagen fibers not only initiate bone mineralization but also support cell adhesion and proliferation [18]. Integrins—key adhesion molecules—regulate osteoblast migration, differentiation, and bone formation [19]. In this study, Gen-OS+TSV and GTO upregulated all investigated integrins, with Gen-OS+TSV showing the strongest effects on α2 and β3 and GTO on α5 and β1. The increased expression of these integrins likely enhances osteoblast adhesion to collagen-based granules combined with TSV gel. Specifically, α2β1 facilitates interaction with collagen I, while α5β3 supports matrix remodeling [20,21], highlighting the biological relevance of collagen in the bone substitutes. After the attachment phase, osteoblasts synthesize extracellular collagenous and non-collagenous matrix proteins [22]. Mineralization occurs through the precipitation of hydroxyapatite (HA), and the process requires interaction with collagen fibrils [10,23,24]. In the present study, all tested collagenic bone grafts promoted collagen secretion after 7 days and calcium deposition after 14 days compared with the control, with the highest values being in the Gen-OS+TSV and GTO groups. The activity of ALP was also stimulated in the Gen-OS+TSV and GTO groups. This result agreed with a previous in vivo and in vitro study. The authors reported that collagenic porcine graft was able to regenerate new bone in critical defects of rabbit calvaria and to promote ALP activity in osteoblasts [25]. ALP in osteoblasts is an important enzyme in the process of biomineralization. Its action in the early stages of differentiation of osteoblasts seems to be responsible for enabling the deposition of hydroxyapatite crystals during bone formation process, hydrolyzing extracellular inorganic pyrophosphate under alkaline conditions [10,26]. The normal process of mineral precipitation, and thus ossification, is primarily dependent upon phosphate concentration, with calcium following secondarily [10]. Regarding the measurement of ions in the extracellular environment, in the present study, the combination of collagenic bone granules with TSV gel (Gen-OS+TSV, GTO) affected the surrounding environment of osteoblasts by increasing calcium and phosphate ions. Interestingly, the extracellular environment containing only biomaterials exhibited considerably higher concentrations of phosphate and calcium ions than when osteoblasts were also present, suggesting a cellular uptake mechanism of these ions. Indeed, the observed higher mineralization, assessed through ALP activity and calcium deposition, in the Gen-OS+TSV and GTO groups could be explained by the rapid augmentation of phosphate and calcium ions levels in the environment surrounding osteoblasts. The bone mineral components, phosphate and calcium, are transported within the cells by specific membrane channels, which do not cotransport hydroxyapatite [27,28]. Subsequently, during mineralization, osteoblasts secrete matrix vesicles containing small calcium phosphate crystal precursors [29]. Therefore, the availability of phosphate and calcium ions in the extracellular environment could facilitate the mineralization process of osteoblasts. Once deposited, the mineral undergoes maturation from crystal precursor to hydroxyapatite, with an alkaline pH being maintained to allow the mineral to precipitate [30]. Since the complex remodeling of bone tissue is extremely sensitive to the local pH values, in this study, the pH of cell media was measured. The presence of bone substitutes Gen-OS+TSV, GTO, and TSV gel made the media more alkaline, while no change was observed with Gen-OS. In agreement with our findings, Galow AM et al. showed that alkaline pH enhances osteoblast proliferation and accelerates calcification [30]. The analysis of osteogenic key mRNA revealed that the expression of BMP-2 was upregulated by all collagenic bone substitutes. It has been demonstrated that BMP-2 can promote the expression of Runx2 in osteoblastic differentiation and can also promote osteoblastic maturation markers such as ALP and OCN [31,32]. In the present study, Runx2 was not upregulated, likely because the osteoblasts used were already in a differentiated state at the time of treatment. As Runx2 is primarily involved in the early stages of osteogenic differentiation, its expression is typically reduced in mature osteoblasts. Meanwhile, the ALP gene was upregulated for all collagenic bone substitutes, mainly for Gen-OS+TSV and GTO. OCN was upregulated only for Gen-OS+TSV and GTO. OCN, which is often used as a marker for the bone formation process, upon its secretion, undergoes a structural change that enables it to bind to calcium in hydroxyapatite. It has been observed that high levels of osteocalcin are associated with increased bone mineral density [33]. Overall, the findings of the present study suggest that TSV gel is not physiologically inert. indeed, it exerted effects on osteoblasts by modulating activities such as adhesion, proliferation, mineralization, collagen deposition, and the expression of osteogenic genes, but above all, when TSV gel was associated with collagenic bone substitutes granules, a stimulation of these activities was observed. Although in the present study the tested bone grafts have different granulometry, the size of granules seems not to affect the osteoblasts as much as the presence of collagen TSV gel. The results suggested that the combination of collagenic biomaterials with TSV gel boosted osteoblasts by creating a suitable microenvironment, enhancing calcium and phosphate ion concentration, and promoting integrin-mediated cell–collagen interaction. Overall, this study highlights some possible biological mechanisms that may lead to further investigations. Understanding the biological processes of bone graft materials and their potential effects can be useful in predicting their clinical outcomes.

## 4. Materials and Methods

### 4.1. Study Design and Bone Substitutes

**Figure 8:**
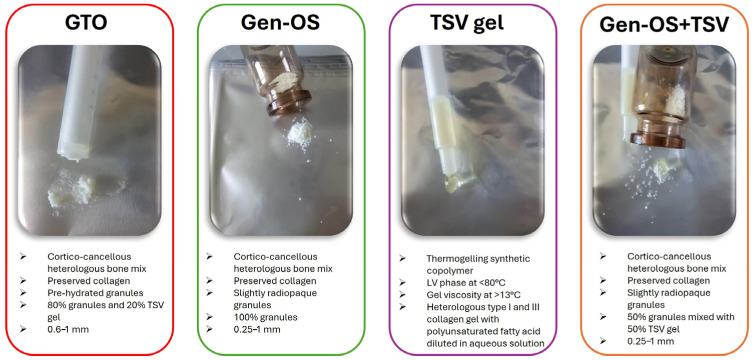
Characteristics of bone substitutes (LV: low viscosity)**.**

In this study, the test groups were osteoblasts exposed to GTO, Gen-OS, TSV-Gel, and Gen-OS+TSV (Figure 8), whereas the unexposed hOBs were the control group (CTRL):

- The GTO group included osteoblasts cultured with OsteoBiol^®^ GTO^®^, composed of 80% xenogeneic collagenic cortico-cancellous granules, ranging in size from 600 to 1000 μm, and 20% TSV gel. OsteoBiol^®^ GTO^®^ was used as the manufacturer with an applicator syringe.
- The Gen-OS group included osteoblasts cultured with OsteoBiol^®^ Gen-OS^®^, composed of xenogeneic collagenic cortico-cancellous granules, ranging in size from 250 to 1000 μm.
- The TSV gel group included osteoblasts cultured with TSV gel, a synthetic copolymer containing type I and III collagen along with polyunsaturated fatty acids. TSV gel was used as the manufacturer with an applicator syringe.
- The Gen-OS+TSV group included osteoblasts cultured with OsteoBiol^®^ Gen-OS^®^ granules mixed with TSV gel for this study at a ratio of 50:50 (%).
- The CTRL group included untreated osteoblasts.

All bone substitutes of the OsteoBiol^®^ series (GTO^®^, Gen-OS^®^, TSV-Gel) tested in this study were obtained from Tecnoss^®^ Dental s.r.l. (Turin, Italy). OsteoBiol^®^ dual-phase collagenic xenografts were manufactured by Tecnoss s.r.l (Giaveno, Italy).

hOBs were placed into the well/plates, cultured with different bone substitutes, and then subjected to the following biological analyses:

i. Cell viability assessment at 24 and 48 h with the 3-(4,5-dimethylthiazol-2-yl)2,5-diphenyltetrazolium bromide (MTT) assay.
ii. Assessment of hOBs adhesion and morphology at 24 and 48 h using Toluidine Blue staining.
iii. Assessment of collagen secretion by hOBs at 7 days using Picro-Sirius Red staining and spectrophotometric analysis.
iv. Assessment of mineralization by hOBs at 14 days with Alizarin Red staining and spectrophotometric quantification and ALP activity at 7 days.
v. Measurement of extracellular phosphatase and calcium ions in culture media at 24 and 48 h by a colorimetric assay.
vi. Measurement of pH in culture media at 24 and 48 h by pH meter.
vii. Assessment of osteogenic markers and integrins gene expression in hOBs at 48 h using real-time quantitative polymerase chain reactions (RT-qPCRs).

### 4.2. Cell Culture

Primary osteoblasts (hOBs) were isolated from mandibular bone fragments of n° 12 patients subjected to dental surgery at the Dental School of University G. d’Annunzio Chieti-Pescara (ethical approval BONEISTO N.22, 7 October 2021). All donors were systemically healthy, non-smokers, and not taking any medication known to affect bone metabolism. These primary cells were isolated using enzymatic digestion [34] from alveolar bone samples collected during routine oral surgical procedures.

The cells were cultured in DMEM low glucose (1 g/L) (SIAL, Rome, Italy) supplemented with 10% fetal bovine serum (FBS), 1% penicillin-streptomycin, and 1% L-glutamine (SIAL). The cells were maintained at 37 °C in a humidified atmosphere with 5% CO_2_.

### 4.3. Viability 1 mg/mL, 5 mg/mL, 10 mg/mL, 24 h, 48 h

To determine the optimal concentration for in vitro experiments, an MTT assay was conducted using bone substitutes at concentrations of 1 mg/mL, 5 mg/mL, and 10 mg/mL. hOBs were cultured in a 24-well plate at a density of 2 × 10^4^ cells/well for 24 h. Then, cells were treated, and after 24 and 48 h, 100 μL of MTT solution (5 mg/mL) (Sigma Aldrich, St. Louis, MO, USA) was added to each well and incubated for 4 h at 37 °C. The formazan crystals were dissolved in 200 μL of DMSO, and absorbance was measured at 570 nm using a microplate reader (SynergyH1 Hybrid BioTek Instruments, Santa Clara, CA, USA).

### 4.4. Toluidine Blue Staining 1 mg/mL, 24 h, 48 h

To evaluate osteoblast morphology, cells were seeded in 24-well plates at a density of 2 × 10^4^ cells/well and cultured for 24 h. hOBs were treated with 1 mg/mL of bone substitutes. After two washes with PBS, adherent cells were fixed with 70% cold ethanol for 15 min. Then, hOBs were stained with 1% Toluidine Blue for 10 min at room temperature and washed with distilled water. The cell density was observed under a stereomicroscope at 18× magnification, and images were captured (Leica, Wetzlar, Germany).

### 4.5. Picro-Sirius 1 mg/mL, 7 Days

hOBs were cultured at a density of 5 × 10^4^ cells/well and, after 24 h, were treated with 1 mg/mL of each bone substitute for 7 days to assess collagen deposition. Cells were fixed in 4% paraformaldehyde and stained with Picro-Sirius Red solution (0.1% Sirius Red in saturated picric acid, Sigma Aldrich) for 1 h. Excess stain was removed by washing with 0.5% acetic acid. Images were captured for analysis under light microscopy (Leica) at a magnification of 25×. To quantify collagen content, Picro-Sirius Red was eluted with 0.1 N sodium hydroxide for one hour, followed by spectrophotometric analysis at a 540 nm wavelength using a microplate reader (Synergy H1 Hybrid, BioTek Instruments).

### 4.6. Alizarin Red Staining, 1 mg/mL, 14 Days

To assess mineralization, 5 × 10^4^ cells/well hOBs were seeded and treated with 1 mg/mL of each bone substitute for 14 days. Cells were fixed with 2.5% glutaraldehyde for 2 h at 4 °C and then stained with Alizarin Red S (pH 4.2) (Sigma Aldrich) for 1 h. Excess dye was washed off with distilled water, and the calcium deposits were observed and imaged under a light microscope (Leica) at 25× magnification. Next, 1 mL of 10% cetylpyridinium chloride (CPC; Sigma-Aldrich) was added to quantify calcium deposits through the chelation of calcium ions. After 1 h of incubation, the absorbance was measured at 540 nm using a microplate reader (Synergy H1 Hybrid; BioTek Instruments).

### 4.7. ALP Activity 1 mg/mL, 7 Days

hOBs were seeded at a density of 5 × 10^4^ cells/well and, after 24 h, were treated with each bone substitute (1 mg/mL). After 7 days of culture, ALP activity was measured using a colorimetric assay (AB83369, Abcam Inc., Cambridge, UK). Cells were lysed through a tissue rupture device (QIAGEN, Hilden, Germany). This solution was centrifugated at 10,000× *g* for 15 min, and the supernatant was collected. Next, the lysates were incubated with p-nitrophenyl phosphate (pNPP) substrate in an alkaline buffer (pH 10.2) at 37 °C for 30 min. The reaction was stopped with 0.5 M NaOH, and absorbance was read at 405 nm.

### 4.8. Phosphate Ions (Extracellular) 1 mg/mL, 24 h, 48 h

The concentration of extracellular phosphate ions was measured at 24 and 48 h post-treatment with 1 mg/mL of each bone substitute, both in the presence and absence of cells. According to the manufacturer, the phosphate content in the culture supernatants was determined using a colorimetric assay (Merck, Darmstadt, Germany). Then, the absorbance was measured at 650 nm with a microplate reader (Synergy H1 Hybrid, BioTek Instruments).

### 4.9. Calcium Ions (Extracellular) 1 mg/mL, 24 h, 48 h

Extracellular calcium ion concentrations were measured at 24 and 48 h after treatment with 1 mg/mL of each bone substitute, both in the presence and absence of cells. The calcium content in the culture supernatants was determined using a colorimetric assay (Merck, Darmstadt, Germany) according to the manufacturer’s instructions. The absorbance was read at 570 nm using a microplate reader (Synergy H1 Hybrid, BioTek Instruments).

### 4.10. pH Measurements 1 mg/mL, 24 h, 48 h

The pH of the culture medium was initially measured in the presence of each bone substitute (at 1 mg/mL) but in the absence of cells to evaluate the direct effect of the biomaterials on the extracellular environment. Measurements were taken at 24 and 48 h post-incubation. Subsequently, the pH of the medium from osteoblast cultures treated with the same concentration (1 mg/mL) of each bone substitute was also measured at the same time points. The measurements were performed by using a calibrated pH meter (HANNA Instruments, Villafranca Padovana, PD, Italy). 

### 4.11. Gene Expression, 1 mg/mL, 48 h

Total RNA was extracted from treated osteoblasts at 48 h using TRIfast reagent (Euroclone, Pero, MI, Italy) according to the manufacturer’s instructions. RNA concentration and purity were determined using a NanoDrop spectrophotometer (Implen NanoPhotometer, Westlake Village, CA, USA). cDNA was synthesized from 1 μg of total RNA using a reverse transcription kit (GoTaq^®^ 2 Step RT-qPCR Kit, Promega, Madison, WI, USA). Gene expression levels of osteogenic markers (ALP, RUNX2, BMP2, OCN) and integrins (integrins α2, α5, β1, β3) were quantified by qRT-PCR (Quant Studio 7 Pro Real-Time PCR System, ThermoFisher, Waltham, MA, USA) using SYBR Green Master Mix (GoTaq^®^ 2 Step RT-qPCR Kit, Promega) (Table 3). β-actin was used for housekeeping. Relative gene expression was calculated using the 2^−ΔCt^ method.

### 4.12. Statistical Analysis

Statistical analysis was performed using GraphPad 8.0.2.263 software (GraphPad, San Diego, CA, USA). The Shapiro–Wilk test was used to assess the normality of the data. Then, a one-way ANOVA test was employed, followed by a post hoc Tukey’s multiple comparisons test, to assess significant differences between groups. Statistical significance was considered with *p* < 0.05. All data were obtained from three independent experiments, each performed in triplicate.

## 5. Conclusions

This study demonstrated that different bone graft materials exert distinct effects on human oral osteoblast activity in vitro. Notably, the combination of TSV gel with collagenic bone graft granules significantly enhanced osteogenic responses, potentially due to integrin-mediated osteoblast–collagen interactions and increased extracellular calcium and phosphate ion availability. While these findings provide valuable preliminary insights, they are based on an in vitro model and should be interpreted with caution. The absence of complex biological interactions present in vivo represents a key limitation. Therefore, further investigations, particularly in vivo studies and clinical trials, are needed to validate the translational relevance of these results.

## Figures and Tables

**Table 1:** pH measured in culture media with biomaterials only. All data were obtained from three independent experiments, each performed in triplicate.

1 mg/mL	CTRL	+GTO	+Gen-OS	+TSV gel	+Gen-OS+TSV
24 h	7.74	8.87	7.72	8.14	9.11
48 h	8.01	8.62	8.07	8.60	8.62

**Table 2:** pH measured in cell culture with osteoblasts treated with biomaterials. All data were obtained from three independent experiments, each performed in triplicate.

1 mg/mL	DMEM	+GTO	+Gen-OS	+TSV gel	+Gen-OS+TSV
24 h	7.51	8.84	7.74	8.65	9.06
48 h	7.46	8.63	8.07	8.60	8.62

**Table 3:** Primer sequences used in RT-qPCR (F: forward; R: reverse).

Gene	Primer Sequences (5’-3’)
*OCN*	F TCAGCCAACTCGTCACAGTC R GGCGCTACCTGTATCAATGG
*RUNX2*	F ACAAGGTGGTCGAAGACTGG R TCTGGTCCTAGTGCCTCACAT
*BMP2*	F GGAAGCAGCAACGCTAGAAG R GACTGCGGTCTCCTAAAGGTC
*ALP*	F AATGAGTGAGTGACCATCCTGG R GCACCCCAAGACCTGCTTTAT
*INTEGRIN α2*	F TCAGGCACACCAAAAGAATTG R CGTCTTTCAACCAGCAGGTAA
*INTEGRIN α5*	F ACATCTGTGTGCCTGACCTG R CCAGGTACACATGGTTCTGC
*INTEGRIN β3*	F GTGTGCCTGCTCTGAT R AGCAGATTCTCCTTCAGGTCA
*β-ACTIN*	F CCAGAGGCGTACAGGGATAG R GAGAAGATGACCCAGGACTCTC

## Data Availability

All the original contributions presented in this study are included in the article. Further inquiries can be directed to the corresponding author.
